# Development and Application of an Antigen Capture ELISA for the Detection of Enzootic Nasal Tumor Virus‐2

**DOI:** 10.1155/tbed/5514208

**Published:** 2025-12-19

**Authors:** Yang Zhao, Jinling Wang, Qiang Liu, Jiang Wu, Qixin Huang, Bingwu Zhang, Yunze Guo, Chang Liu, Xing Guo, Kui Guo, Weiguo Zhang, Xiaohua Ma, Xue-Feng Wang, Xiaojun Wang

**Affiliations:** ^1^ State Key Laboratory of Animal Disease Control and Prevention, Harbin Veterinary Research Institute, The Chinese Academy of Agricultural Sciences, Harbin, 150069, China, caas.cn; ^2^ College of Veterinary Medicine, Inner Mongolia Agricultural University, Hohhot, 010018, China, imau.edu.cn; ^3^ Nanchong Key Laboratory of Disease Prevention, Control and Detection in Livestock and Poultry, Nanchong Vocational and Technical College, Nanchong, 637131, China; ^4^ College of Coastal Agricultural Sciences, Guangdong Ocean University, Zhanjiang, 524088, China, gdou.edu.cn; ^5^ Haixi Center of Animal Disease Prevention and Control, Haixi Mongolian and Tibetan Autonomous Prefecture, 817099, China; ^6^ Dazhou Vocational and Technical College, Dazhou, 635001, China; ^7^ College of Veterinary Medicine, Henan University of Animal Husbandry and Economy, Zhengzhou, 450046, China, hnuahe.edu.cn; ^8^ College of Animal Science, Anhui Science and Technology University, Fengyang, 233100, China, aust.edu.cn; ^9^ Institute of Western Agriculture, The Chinese Academy of Agricultural Sciences, Changji, 831100, China, caas.cn

## Abstract

Enzootic nasal tumor virus (ENTV) is the etiological agent responsible for enzootic nasal adenocarcinoma (ENA), a chronic and contagious disease predominantly affecting sheep and goats. ENTV is classified into two distinct types: ENTV‐1, which infects sheep, and ENTV‐2, which infects goats. ENA has been globally reported in small ruminant‐rearing regions, causing significant mortality and substantial economic impacts in affected flocks. There is currently no standardized detection method for ENA. In this study, we successfully generated a monoclonal antibody (mAb) and a polyclonal antibody (pAb) against the ENTV‐2 capsid protein (p27), and identified the epitope of the mAb, which was found to be highly conserved among different ENTV‐2 isolates. An antigen capture ELISA (acELISA) was then successfully developed using the mAb as the capture antibody and the pAb as the detection antibody to specifically detect p27 of ENTV‐2 in nasal secretions. The cut‐off value of the acELISA was determined to be 0.1052 by analyzing S/P values. The detection limit of this assay was 0.16 ng/mL of rp27 protein and equivalent to 844 copies/μL of ENTV‐2 RNA. Specificity tests showed that the method had no cross‐reaction with other prevalent small ruminant pathogens. The coincidence rates of the developed acELISA compared with western blotting and qRT‐PCR assays were 98.95% (189/191) and 96.34% (184/191), respectively. Furthermore, the acELISA was applied to assess ENTV‐2 in 1228 clinical nasal swab samples collected from seven provinces in China. The results demonstrated that the positivity rate varied between 0.00% and 28.21%. In conclusion, we successfully developed an acELISA with high specificity, sensitivity and reproducibility for the detection of ENTV‐2 antigen. This high‐throughput method for the detection of ENTV‐2 represents a significant advancement in the field and may contribute to the prevention and control of ENTV‐2.

## 1. Introduction

Enzootic nasal adenocarcinoma (ENA), also known as enzootic nasal tumor (ENT), is characterized by tumoral proliferation of epithelial cells in the nasal cavity and is a chronic contagious disease of sheep and goats caused by the enzootic nasal tumor virus (ENTV) [1]. ENTV has not yet been classified by the International Committee on Taxonomy of Viruses (ICTV). The ENTV genome is a single‐stranded positive‐sense RNA of ~7.5 kb in length [2] and has high homology to jaagsiekte sheep retrovirus (JSRV), a member of the genus *Betaretrovirus* of the family *Retroviridae*, which causes neoplastic transformation of alveolar and bronchiolar secretory epithelial cells resulting in lung tumors in sheep and goats, known as ovine pulmonary adenocarcinoma (OPA). There are two types of ENTV: ENTV‐1, which infects sheep, and ENTV‐2, which infects goats. These three retroviruses (ENTV‐1, ENTV‐2 and JSRV) share more than 88% sequence identity [3, 4]. ENA and OPA usually have long incubation periods, but both diseases are fatal due to the development of tumors in infected animals, loss of body weight, and concomitant bacterial infections that ultimately lead to pneumonia or septicemia, making both diseases a serious threat to the development of the sheep and goat industry.

Similar to JRSV, ENTV also has four overlapping genes: *gag*, *pro*, *pol*, and *env*. The *gag* gene encodes a Gag precursor protein that is cleaved by viral proteases into the matrix protein (MA), capsid protein (CA, p27), and nucleocapsid protein (NC). The *pro* gene encodes viral protease (PR) that is involved in the proteolytic processing of viral proteins. The *pol* gene encodes reverse transcriptase (RT) and integrase (IN), which are essential for viral replication and are involved in reverse transcription and integration of the genome, respectively. The *env* gene encodes the surface glycoproteins (SU) and transmembrane protein (TM) that are involved in viral entry into host cells [5, 6]. CA is the most abundant component of the retroviral virion and is highly conserved within each species of virus. Therefore, CA is an important biomarker for the etiological detection of retroviral infection and replication [7–9].

ENA occurs naturally in all continents except Australia and New Zealand [10]. It is clinically characterized by continuous nasal fluid discharge, respiratory distress, exophthalmos, and skull deformations and has a high mortality rate [11], resulting in significant economic losses to goat and sheep flocks. In China, ENTV‐2 infections have been reported in several provinces in recent years [12–15], but ENTV‐1 infections have not. At present, diagnosis of ENA is routinely accomplished through assessment of clinical signs, histopathology, conventional reverse transcription‐polymerase chain reactions (RT‐PCR), and real‐time quantitative RT‐PCR (qRT‐PCR) [16–18]. ENT cannot be diagnosed by virus isolation because there are no in vitro cell culture systems for ENTV. Serological tests have not been used for the diagnosis of ENA due to the lack of evidence of circulating antibodies against ENTV in infected animals, with only one laboratory reporting the presence of antibodies against Env in some ENTV‐1‐infected sheep [19].

Enzyme‐linked immunosorbent assay (ELISA) is known for its low cost and high throughput, making it the preferred method for disease detection and conducting large‐scale surveillance of many infectious diseases. In this study, we generated a monoclonal antibody (mAb) and a polyclonal antibody (pAb) against ENTV‐2 p27 and developed an antigen capture ELISA (acELISA) using this antibody pair. Furthermore, we evaluated the sensitivity and specificity of this acELISA and compared its agreement with qRT‐PCR and western blotting (WB). The acELISA was further used to investigate the prevalence of ENTV‐2 in goat herds from some areas of China.

## 2. Materials and Methods

### 2.1. Cells, Vector, and Animals

The SP2/0 myeloma cells were cultured in RPMI‐1640 (Sigma–Aldrich, USA) supplemented with 10% heat‐inactivated fetal bovine serum (FBS). HEK293T (human embryonic kidney 293T) cells were maintained in Dulbecco’s Modified Eagle Medium (DMEM; Thermo Fisher Scientific, USA) supplemented with 10% heat‐inactivated FBS. Cells were cultured at 37°C in a humidified atmosphere containing 5% CO_2_. The eukaryotic expression plasmids pcDNA‐ENTV‐1‐Gag, pcDNA‐ENTV‐2‐Gag, and pcDNA‐JSRV‐Gag, encoding ENTV‐1 Gag, ENTV‐2 Gag, and JSRV Gag respectively, were synthesized and codon‐optimized by SyngenTech (Beijing) Biotechnology Co., Ltd. The small ruminant lentiviruses (SRLV) Gag expression plasmids pcDNA‐SRLV‐Gag‐HA have been reported previously [20]. The prokaryotic expression plasmids pGEX‐6P‐ENTV‐1‐p27 and pGEX‐6P‐JSRV‐p27, which encode ENTV‐1 p27 and JSRV p27, respectively, were synthesized by SyngenTech (Beijing) Biotechnology Co., Ltd. These constructs were used to purify recombinant ENTV‐1 p27 and JSRV p27 proteins following a previously described method [20]. The prokaryotic expression vectors pET‐30a (+) and pET‐32a (+) were maintained in our laboratory. Eight‐week‐old SPF grade BALB/c female mice and 4‐month‐old female New Zealand white rabbits were provided by the Animal Experiment Center of Harbin Veterinary Research Institute.

### 2.2. Preparation of Recombinant ENTV‐2 p27 Protein

The proviral DNA was extracted from the tumor tissue of goats with ENTV‐2 using a TIANamp Genomic DNA Kit (TIANGEN BIOTECH CO., Ltd., Beijing, China) [15]. The complete p27 gene was amplified with the primers ENTV‐p27‐F (5′‐GGAATTCTTTAAGCAATTAAAAGAGTTAAAG‐3′) and ENTV‐p27‐R (5′‐CGCTCGAGTTATCCAATATCAGCGCAGATGCGAAT‐3′) using the proviral DNA as a template. The *p27* gene was then cloned into the *EcoR* I and *Xho* I restriction sites of the pET‐30a (+) and pET‐32a (+) expression vectors, and the recombinant plasmids (pET‐30a‐p27 and pET‐32a‐p27) were transformed into *E. coli* BL21 (DE3), followed by induction at 25°C for 12 h in the presence of 0.5 mM isopropyl‐*β*‐D‐thiogalactoside (IPTG) when the OD_600_ nm of the bacterial culture reached ~0.6. The cultures were collected by centrifugation at 6,000×*g* for 5 min at 4°C, followed by resuspension of the pellet in 40 mL binding buffer (pH 8.0). The transformed pET‐30a‐p27 cells were sonicated, and recombinant ENTV‐2 p27 (rp27) was purified using High Affinity Ni‐Charged Resin (GenScript, China) according to the manufacturer’s instructions. The pET‐32a (+) expression vector contains not only a His tag but also a Trx tag comprising 109 amino acids. In this study, the pET‐32a‐p27 served as the template for constructing truncated p27 expression vectors. These mutant constructs were subsequently utilized in epitope mapping experiments, as detailed in Section 2.5.

### 2.3. Preparation and Identification of mAb Against ENTV‐2 p27

8‐week‐old female BALB/c mice were immunized with purified rp27 that had been emulsified using Freund’s complete adjuvant. Fourteen days after the initial immunization, mice were immunized again with purified rp27 emulsified with Freund’s incomplete adjuvant every 14 days. After four immunizations, splenocytes from the immunized mice were obtained and fused with SP2/0 myelomas in PEG solution (Sigma–Aldrich, Germany). Hybridoma cells were maintained in HAT (Hypoxantin, Aminopterin, Thymidin) medium, which was gradually replaced with HT (Hypoxanthine, Thymidine) medium for the screening of hybridoma cells. The supernatant of hybridoma cells was harvested, and the efficacy of the antibodies against the rp27 was tested using indirect ELISA (iELISA). Positive hybridoma cells were screened three times through subcloning, and the monoclonal cells that stably secreted the specific antibody against rp27 were selected for subsequent iELISA experiments. Freund’s incomplete adjuvant (500 μL) was injected into each BALB/c mouse. 1 week later, 1 × 10^6^ hybridoma cells were injected into the abdominal cavity of each BALB/c mouse to produce ascites, and ascites were collected daily with a sterile syringe 1 week after injection of hybridoma cells. The collected ascites were centrifuged at 4°C to remove impurities, and the supernatant was collected. The monoclonal antibodies were purified using Protein G resin (GenScript, China) according to the manufacturer’s instructions.

### 2.4. Generation of pAb Against ENTV‐2 p27 in Rabbits

Four‐month‐old female New Zealand white rabbits were immunized with the purified rp27 emulsified using Freund’s complete adjuvant. The rabbits were immunized subcutaneously with an emulsion containing rp27 at five sites per rabbit (0.2 μg per site). Pre‐immune serum samples to serve as the negative controls were collected from the marginal ear vein of the rabbits under sterile conditions. Fourteen days following initial immunization, rabbits were immunized with purified rp27 emulsified using Freund’s incomplete adjuvant, and immunization was repeated every 14 days. After four immunizations, blood samples were collected using cardiac puncture. The sera obtained from the blood samples were then stored at −80°C until needed for further analysis.

### 2.5. Mapping the Epitope Recognized by mAb 2C3

To identify the epitope recognized by the mAb 2C3, a series of expression plasmids for truncated p27 mutants were constructed using pET‐32a (+) as the vector. First, the expression plasmids for three overlapping truncated fragments (P1, residues 1–77; P2, residues 65–138; P3, residues 128–197) of the ENTV‐2 p27 were constructed using the pET32a‐p27 as a template via PCR mutagenesis. Then, the expression plasmids for two overlapping p27 fragments (P3A, residues 128–166; P3B, residues 157–197) were constructed using the P3 expression plasmid as a template via PCR mutagenesis. Finally, the expression plasmids for more progressively truncated p27 fragments were constructed using the P3B expression plasmid as a template via PCR mutagenesis. The *E. coli* BL21(DE3) cells with different plasmids were induced to express by IPTG. The reactivity between 2C3 and the expressed recombinant proteins was analyzed with WB. The experimental procedures were performed in accordance with established protocols as previously described [20]. 2C3 served as the primary antibody, and anti‐His antibodies were utilized as the loading control. Anti‐mouse IgG‐DyLight 800 was employed as the secondary antibody, and membranes were visualized using the LI‐COR Odyssey Imaging System (LI‐COR, Lincoln, NE, USA).

To assess the degree of conservation of the E177−194 epitope, 35 ENTV‐2 p27 amino acid sequences were obtained from Genbank (Supporting Information: Table S1), multiple alignments of the E177−194 amino acid sequences were performed using the Clustal W method within DNASTAR software version 7.0, and then a WebLogo diagram was constructed using the TBtools‐II software.

### 2.6. Development of the acELISA Method

2C3 was used as the capture antibody, and pAb‐p27 was used as the detection antibody. The coating concentration of 2C3 (4, 2, 1, and 0.5 μg/mL) and the dilution of pAb‐p27 (1:250,000, 1:500,000, 1:1,000,000 and 1:2,000,000) were optimized using checkerboard titration. The procedure was performed as follows: first of all, different concentrations of 2C3 (diluted in PBS) were coated with 100 μL overnight at 4°C. After washing three times with PBS containing 0.05% Tween‐20 (PBST), the plates were blocked with 200 µL of blocking buffer (5% skimmed milk powder in PBS, pH 7.4) for 2 h at 37°C. After three washes, 100 μL purified rp27 protein (5 ng/mL in 5% skimmed milk/PBS) was added to the wells and incubated for 2 h at 37°C. Following a further three washes, 100 μL diluted pAb‐p27 (diluted in 5% skimmed milk/PBS) was added to each well, and the plates were incubated for 2 h at 37°C. The plates then were washed three times with PBST. A 1:5000 dilution of 100 μL HRP‐conjugated goat anti‐rabbit immunoglobulin G (ZSGB‐BIO, China) was then added to each well and incubated for 1 h at 37°C. Following incubation, the plates were washed three times and inoculated with 3,3′,5,5′‐tetramethylbenzidine (TMB) substrate for 10 min. This reaction was stopped by the addition of 2MH_2_SO_4_, and the optical density (OD) values were measured at 450 nm using the VersaMax Microplate Reader (BioTek, Winooski, VT, USA).

### 2.7. Optimization of the acELISA Reaction Conditions

To establish the acELISA assay, the blocking buffer, blocking time, incubation time of antigen, detection antibody and HRP‐labeled IgG were optimized. Blocking time and incubation time (antigen and detection antibody) both ranged from 1 to 2 h. The incubation time of HRP‐labeled IgG ranged from 30 to 60 min. The blocking buffers were selected from 2% and 5% skimmed milk, 2% and 5% bovine serum albumin (BSA), 2% and 5% NH_4_Cl, 2% and 5% gelatin. The sample dilution was used as a negative control, and the optimal reaction conditions were determined based on a ratio of OD values of the p27 protein to the negative control (P/N).

### 2.8. Determination of the Cut‐Off Value for the acELISA

A total of 105 nasal swab samples were collected from four different goat farms and verified as either ENTV‐negative or ‐positive by WB. Among these, 74 samples were confirmed as ENTV‐2 negative and originated from three farms where no ENTV‐2 positive cases were detected. The remaining 31 were ENTV‐2 positive samples collected from a goat farm in which an ENTV‐2 infection was occurring. For WB analysis, mAb 2C3 was used as the primary antibody (at a 1:2000 dilution), and anti‐mouse IgG conjugated with DyLight 800 served as the secondary antibody (at a 1:5000 dilution).

The S/P values of these negative and positive samples were calculated to determine the cut‐off value of the acELISA. The S/P value was calculated using the following formula: S/P value = (sample OD value − negative control OD value)/(positive control OD value − negative control OD value).

### 2.9. Assessment of Specificity, Sensitivity, and Repeatability of the acELISA

The following 10 pathogens were used to assess the specificity of the acELISA method: foot‐and‐mouth disease virus (FMDV, inactivated vaccine; types O and A; The Spirit Jinyu Biological Pharmaceutical Co., Ltd., Inner Mongolia, China), caprine *Streptococcus* (inactivated vaccine; Harbin Pharmaceutical Group Bio‐vaccine Co., Ltd., Harbin, China), *Mycoplasma mycoides* subsp. *capri* (inactivated vaccine; Harbin Pharmaceutical Group Bio‐vaccine Co., Ltd.), Caprine *Escherichia coli* (inactivated vaccine; Harbin Pharmaceutical Group Bio‐vaccine Co., Ltd.), orf virus (ORFV, live vaccine; Shandong Huahong Biological Engineering Co., Ltd., Shandong, China), goat pox virus (GTPV, live vaccine; Harbin Pharmaceutical Group Bio‐vaccine Co., Ltd.), peste des petits ruminants virus (PPRV, live vaccine; Tecon Pharm Co., Ltd., Xinjiang, China), and Gag proteins of ENTV‐1, JSRV, and SRLV were prepared by transfecting the corresponding expression plasmids into HEK293T cells as previously described [20].

A single ENTV‐2 positive nasal swab sample and rp27 were separately and continuously twofold‐diluted in PBS and tested to determine the sensitivity of the acELISA method. The detection limit of the acELISA was based on the cut‐off value. The nasal swab sample was quantified by a previously reported qRT‐PCR assay [17].

To evaluate the repeatability of the assay, five different concentrations of rp27 were measured using five ELISA plates, with three replicates for each concentration, and the inter‐plate coefficient of variation (CV) was calculated. For the intra‐plate CV, five replicates for each concentration within the same ELISA plate were measured. The CV was calculated as (standard deviation/mean value) × 100%.

### 2.10. Quantitative Real‐Time Reverse‐Transcription PCR (qRT‐PCR)

In this study, we employed a previously reported qRT‐PCR assay for the detection of ENTV‐2 [17]. Briefly, viral RNA was extracted from the nasal fluid samples collected from goats, using the TIANamp Virus RNA kit (TIANGEN BIOTECH CO., Ltd., Beijing, China). The extracted RNA was reverse‐transcribed using the HiScript II Q RT SuperMix for qPCR (+gDNA wiper) kit (Vazyme, Nanjing, China). Then real‐time PCR was conducted with TB Green Premix Ex Taq (TaKaRa, Beijing, China) with the primers ENTV‐qPCR‐F (5′‐GAGGCAAATTGAGGCGTTGAT‐3′) and ENTV‐qPCR‐R (5′‐CCCGTTCTGCATTCGCTGTAG‐3′). The reaction mixture (20 μL final volume) consisted of 10 μL TB Green Mix, 1 μL of each primer, 1 μL cDNA, and 7 μL RNase‐free water. PCR amplification was carried out under the following two‐step cycling conditions: initial denaturation at 95°C for 2 min, followed by 40 cycles of 95°C for 5 s and 60°C for 30 s. After amplification, a melting curve analysis was performed to verify the specificity of the amplified products. The protocol included heating to 95°C for 5 s, cooling to 65°C for 5 s, and then gradually increasing the temperature to 95°C while collecting the fluorescence signal.

### 2.11. Clinical Samples

To investigate the prevalence of ENTV‐2 in China, a total of 1228 nasal swab samples were collected from seven different provinces and tested using the established acELISA method. The sample collection procedure and storage are as follows: following proper restraint of the animal, nasal secretions were collected using sterile swabs inserted ~5–10 cm into the nasal passage with gentle rotation for 10 s to ensure adequate mucosal contact. The collected specimens were immediately placed in 3 mL viral transport medium (YOCON, China) and securely sealed in labeled virus sampling tubes.

A total of 100 serum samples were collected from a goat farm in where ENTV‐2‐positive cases had been detected through pathological examination and RT‐PCR [15].

All samples were maintained at 2–8°C during transport using ice‐packed insulated containers to preserve sample integrity. Upon laboratory receipt, 1 mL aliquots of the transport medium were prepared and stored at −80°C for subsequent analyses.

### 2.12. Statistical Analysis

Statistical analysis and data visualization were carried out using GraphPad Prism8 (GraphPad Software, USA). The area under the receiver operating characteristic (ROC) curve (AUC) was employed to assess the accuracy of the tests distinguished between non‐informative (AUC = 0.5), less accurate (0.5 < AUC ≤ 0.7), moderately accurate (0.7 < AUC ≤ 0.9), highly accurate (0.9 < AUC < 1), and perfect tests (AUC = 1) [21].

## 3. Results

### 3.1. Prokaryotic Expression and Purification of Recombination ENTV‐2 p27

Previously, three goats with nasal tumors from a flock were diagnosed with ENTV‐2 infection via pathological examination and RT‐PCR (Supporting Information: Figure S1) [15]. In this study, the complete ENTV‐2 *p27* gene sequence was amplified with PCR from the tumour tissue of one of the goats and then directionally cloned into a pET‐30a (+) vector. Positive recombinant plasmid pET‐30a‐p27 was confirmed with digestion with the *EcoR* I and *Xho* Ⅰ restriction enzymes (Figure 1A) and sequencing. The pET‐30a‐p27 was transformed into *E. coli* BL21 (DE3) competent cells, and the expression of the recombinant protein His‐p27 was induced by the addition of IPTG (Figure 1B) and confirmed with WB analysis using anti‐His antibody (Figure 1C). Subsequently, the recombination p27 protein (rp27) was purified using a Ni‐NTA resin column.

Figure 1Cloning and expression of ENTV‐2 p27. (A) Identification of recombinant expression vector pET30a‐p27 using EcoR Ⅰ/Xhol Ⅰ digestion. (B) SDS‐PAGE analysis of recombinant His‐p27 expression in *E. coli* BL21 (DE3). Lane M, protein marker; lanes 1 and 2, whole cell lysates and supernatant of *E. coli* BL21 (DE3) cell lysates transformed with the empty vector pET30a, respectively; lanes 3 and 4, p27 protein expressed in whole cell lysates of *E. coli* BL21 without and with IPTG induction, respectively. (C) Western blot analysis of recombinant His‐p27 using anti‐His antibody. Lanes 1–4 correspond to the same samples as described for panel B.

**Figure:**
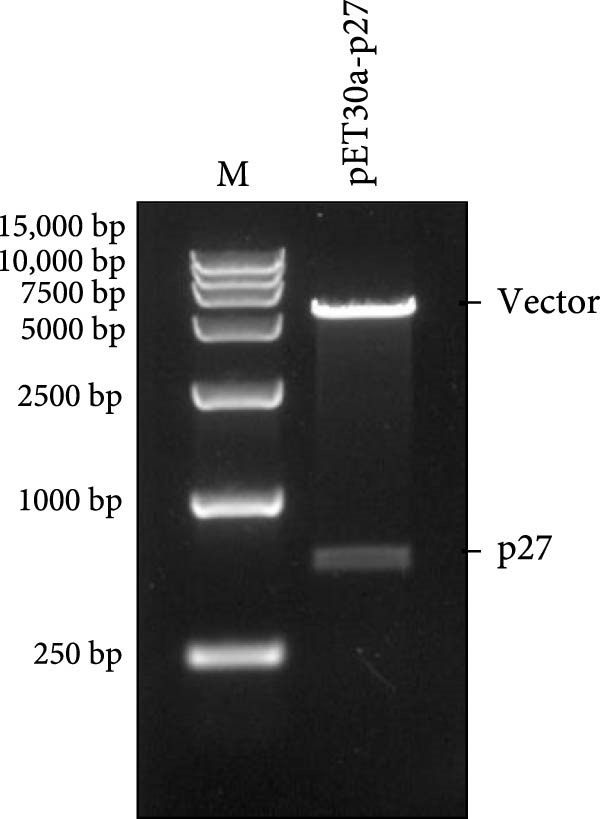
(A)

**Figure:**
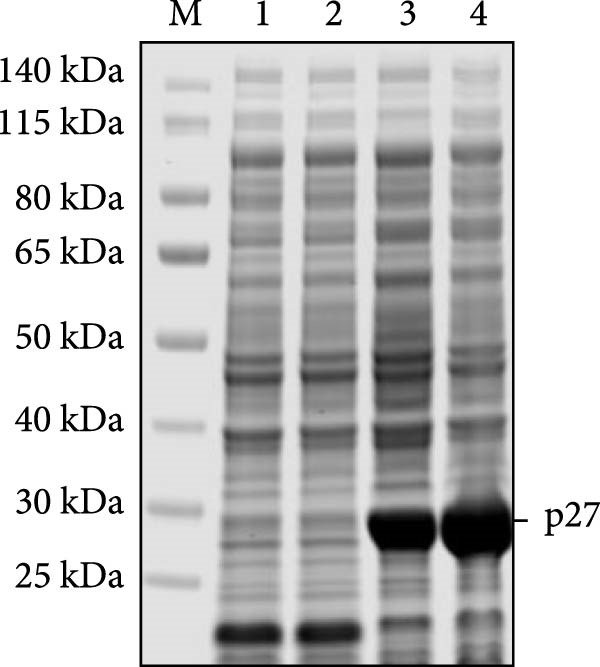
(B)

**Figure:**
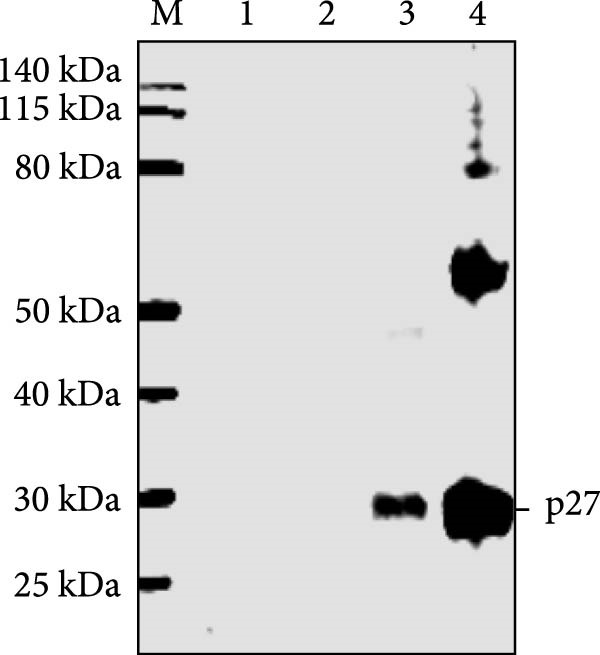
(C)

### 3.2. Preparation of Mouse‐Derived mAb and Rabbit‐Derived pAb Against ENTV‐2 p27

MAbs against the p27 of ENTV‐2 were prepared using hybridoma technology as described in the Materials and Methods section. One stable hybridoma producing mAb against ENTV‐2 p27 protein was obtained and was named 2C3. 2C3 was purified from the ascites fluid of mice injected with hybridoma cells and was subsequently assessed using SDS‐PAGE. The heavy and light chains of the 2C3 were observed at ~53 kDa and 25 kDa, respectively (Figure 2A). The isotypes of 2C3 were identified as IgG1 with the kappa light chain using a commercial kit (Figure 2B).

Figure 2Characterization of 2C3 and pAb‐p27. (A) Identification of purified mAb 2C3 with SDS‐PAGE. Lane M, protein marker; lane 1, mAb 2C3 pre‐purification; lane 2, mAb 2C3 post‐purification. (B) Identification of the 2C3 isotype using a commercially available antibody subtype identification kit. (C) An iELISA was performed using purified rp27 as the coating antigen to detect the serially diluted 2C3, with serum from an unimmunized mouse used as a negative control (NC). The dotted line indicates the cut‐off value, defined as 2.1 times the OD_450_ value of the NC. (D) An iELISA was performed using purified rp27 as the coating antigen to detect the serially diluted pAb‐p27, with serum from unimmunized rabbit used as a NC. The dotted line indicates the cut‐off value, defined as 2.1 times the OD_450_ value of the NC. (E) WB with 2C3 or pAb‐p27 was performed to assess lung, spleen, kidney, lymph and tumor from a goat infected with ENTV‐2. (F) WB with 2C3 or pAb‐p27 was performed to assess nasal secretions from a goat infected with ENTV‐2. NSB, nasal swab. (G) Reactivity of 2C3 or pAb‐p27 with eukaryotic ENTV‐2 Gag protein. HEK293T cells were transfected with pcDNA‐Gag‐HA or empty vector (as a NC). The cells were lysed at 48 hpt for the detection of Gag using 2C3 or pAb‐p27 as primary antibodies.

**Figure:**
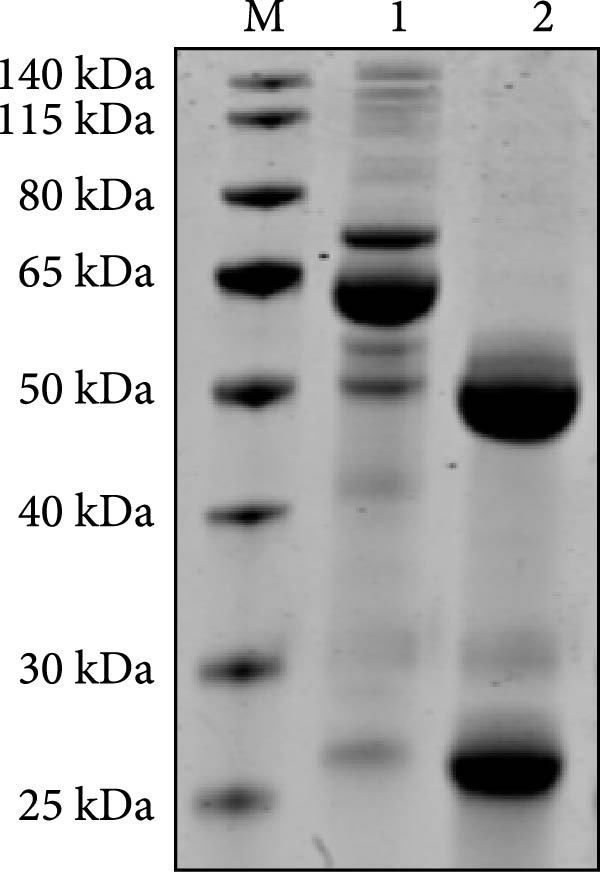
(A)

**Figure:**
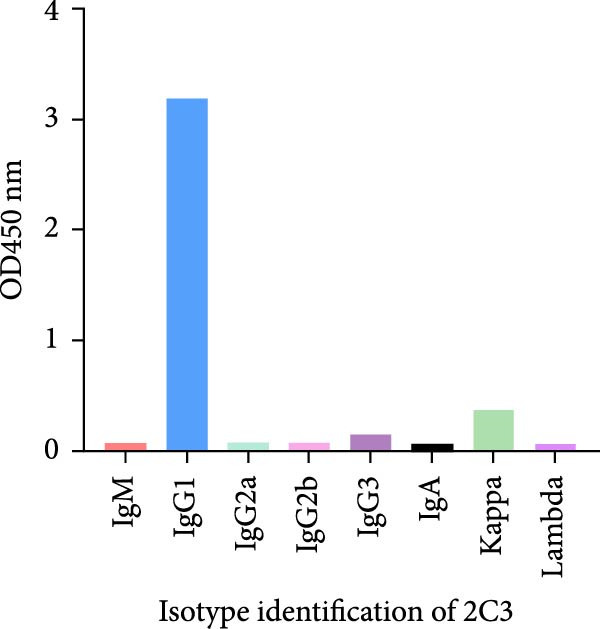
(B)

**Figure:**
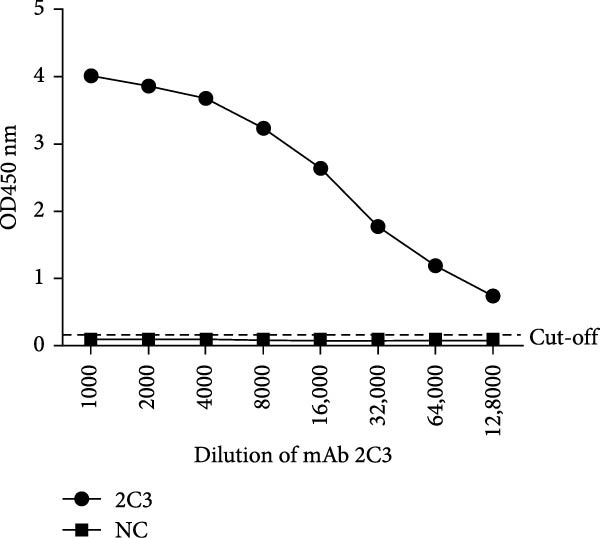
(C)

**Figure:**
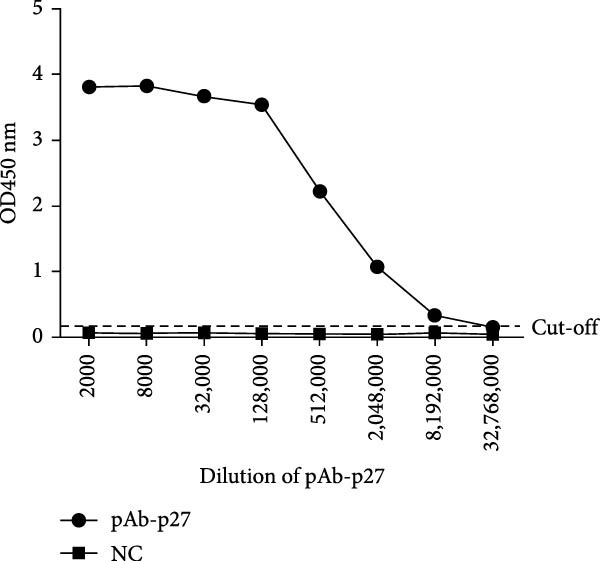
(D)

**Figure:**
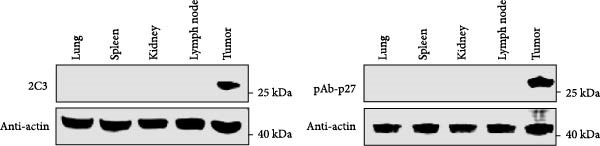
(E)

**Figure:**
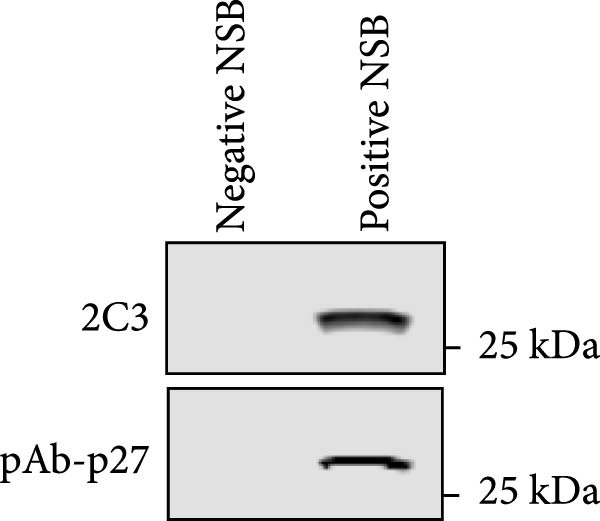
(F)

**Figure:**
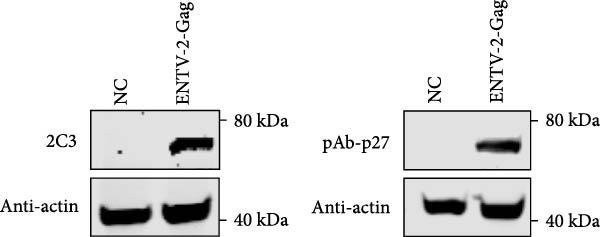
(G)

A rabbit polyclonal antibody against ENTV‐2 p27 (pAb‐p27) was prepared as described in the Materials and Methods. We then performed an iELISA to evaluate the binding affinity of the 2C3 and the pAb‐p27 for the rp27. Serial dilution analysis demonstrated that both 2C3 and pAb‐p27 exhibited exceptional reactivity for ENTV‐2 p27 (Figure 2C,D). WB assay showed that both 2C3 and pAb‐p27 effectively detected p27 in tumor tissue and nasal secretions collected from an ENTV‐2‐infected goat [15] (Figure 2E,F), as well as eukaryotically expressed ENTV‐2 Gag protein (Figure 2G).

### 3.3. Mapping the Epitope Recognized by mAb 2C3

Mapping of the epitope recognized by 2C3 was performed with three overlapping truncated fragments of ENTV‐2 p27, named P1 (1–77aa), P2 (65–138aa) and P3 (128–197aa) (Figure 3A). WB assays demonstrated that 2C3 recognized the P3 fragment (Figure 3B). To further characterize the epitope recognized by the 2C3, we performed several rounds of truncating mutagenesis based on P3B (Figure 3C) and finally confirmed that the minimal linear epitope recognized by the 2C3 was ^177^LRPYRKKGDLSDFIRICA^194^ (named E177−194) (Figure 3D). We next assessed the conservation of E177−194 among ENTV‐2 isolates. A multiple sequence alignment based on 35 ENTV‐2 p27 amino acid sequences containing the E177−194 epitope showed that E177−194 is highly conserved among different isolates of ENTV‐2 (Figure 3E). Subsequently, we performed a protein–protein blast using the LRPYRKKGDLSDFIRICA motif, which revealed that the motif is present not only in ENTV but also in JRSV, as well as in hypothetical proteins of both *Ovis aries* and *Capra hircus* (Supporting Information: Table S2). The results suggest that 2C3 has the potential to be used in the detection of ENTV or JSRV. Indeed, we used WB to demonstrate that both 2C3 and pAb‐p27 also recognized eukaryotically expressed ENTV‐1 and JRSV Gag (Supporting Information: Figure S2).

Figure 3Mapping of the epitope recognized by mAb 2C3. (A) Schematic representation of the p27 fragments used for epitope mapping. The blue lines represent p27 fragments that react with 2C3 and the gray lines represent p27 fragments that do not react with 2C3. The numbers represent the positions of the amino acids in p27. (B) As shown in panel A, several truncated p27 fragments were cloned into pET32a and expressed as His fusion proteins. His‐p27 was segmentally expressed in *E. coli* BL21 (DE3) and then subjected to WB using 2C3 as the primary antibody. (C) Identification of the minimal epitope recognized by 2C3. The blue lines represent p27 fragments that react with mAb 2C3 and the gray lines represent p27 fragments that do not react with 2C3. (D) The reactivity of His‐tagged p27 truncated fragments with the 2C3 analyzed using WB. (E) WebLogo presentation of the variability of epitope E177−194 based on the alignment of 35 amino acid sequences of ENTV‐2 p27 downloaded from the NCBI database.

**Figure:**
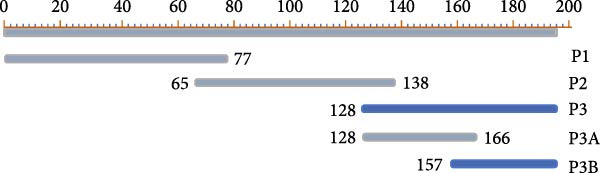
(A)

**Figure:**
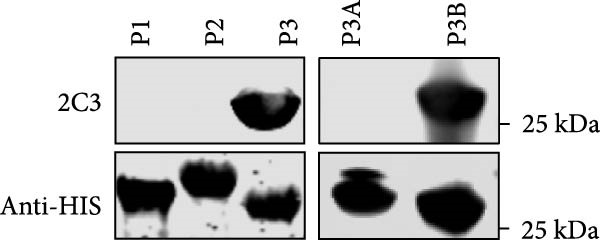
(B)

**Figure:**
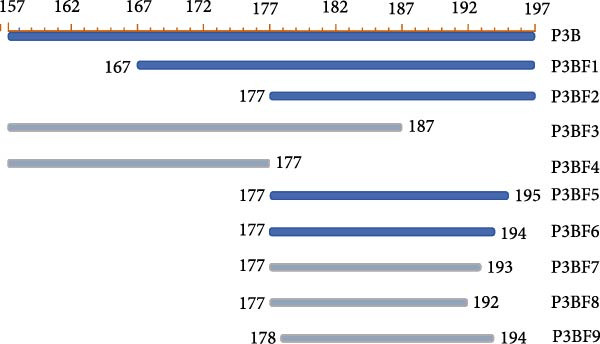
(C)

**Figure:**
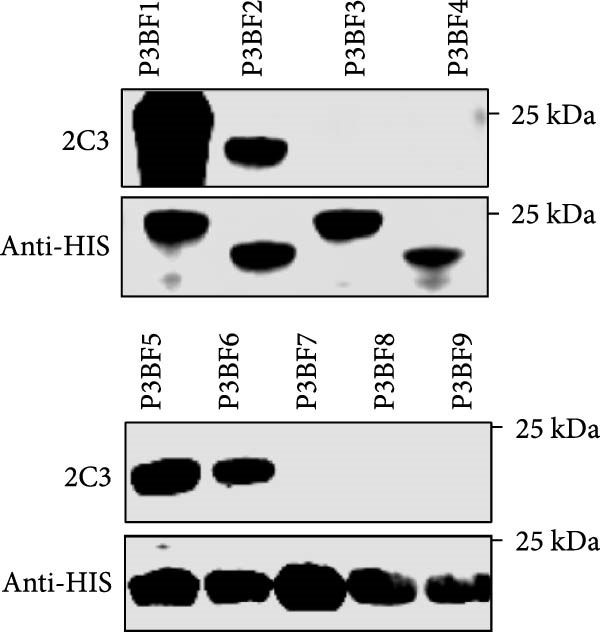
(D)

**Figure:**
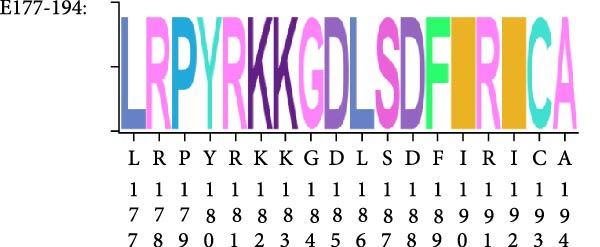
(E)

### 3.4. Establishment and Optimization of the p27‐acELISA

In order to establish an acELISA based on 2C3 and pAb‐p27 (p27‐acELISA) for the detection of ENTV‐2, the optimal working conditions of the p27‐acELISA were optimized with checkerboard titration based on the maximum P/N values. As shown in Table 1, the optimal dosage of the capture antibody 2C3 was 100 ng/well and the optimal dilution of the detection antibody pAb‐p27 was 1:250,000. Using 5% skimmed milk for 1 h had the best blocking efficiency (Figure 4A,B). The optimal incubation time of the antigen was 1.5 h (Figure 4C), the optimal incubation time of the detection antibody was 2 h (Figure 4D), and the optimal incubation time of HRP‐labeled IgG was 45 min (Figure 4E).

Figure 4Optimization of p27‐acELISA reaction conditions. (A) Optimization of blocking buffer. (B) Optimization of the blocking time. (C) Optimization of incubation time for the antigen. (D) Optimization of the incubation time for the detection antibody. (E) Optimization of the incubation time for HRP‐labeled IgG.

**Figure:**
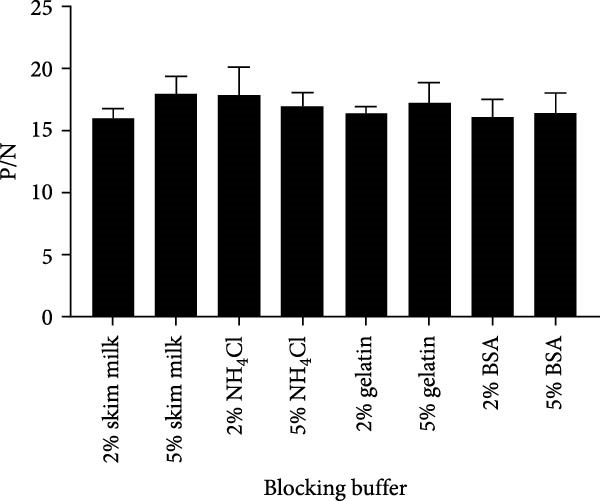
(A)

**Figure:**
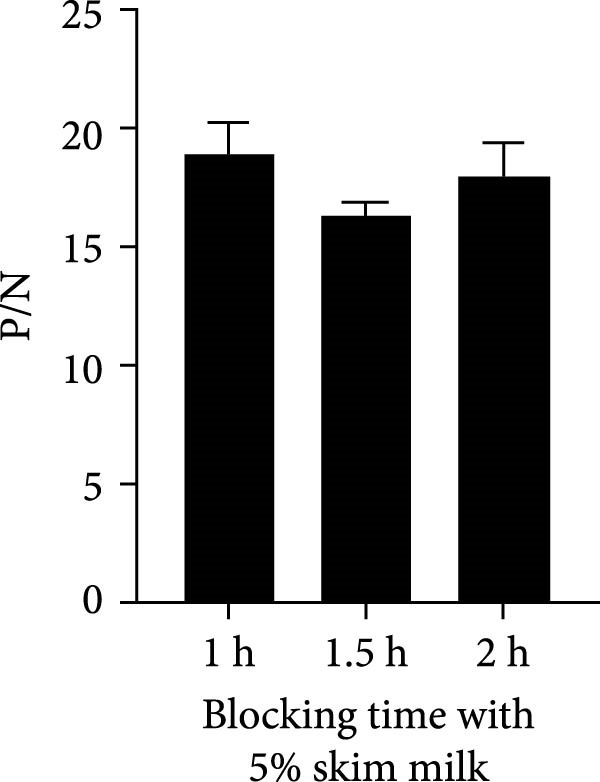
(B)

**Figure:**
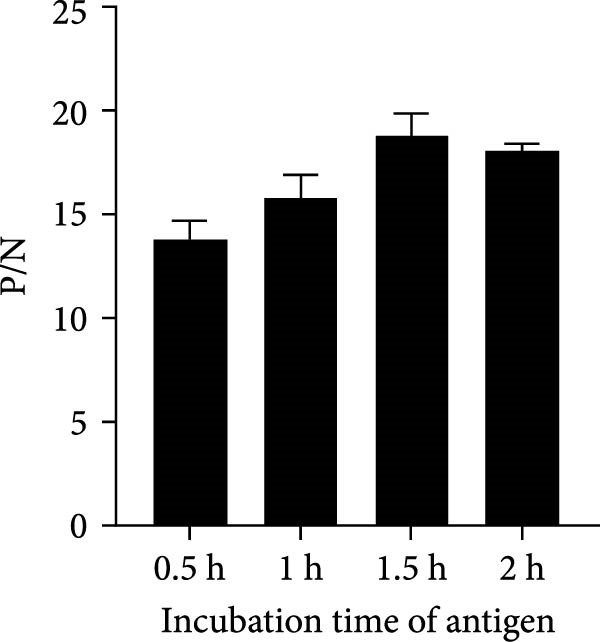
(C)

**Figure:**
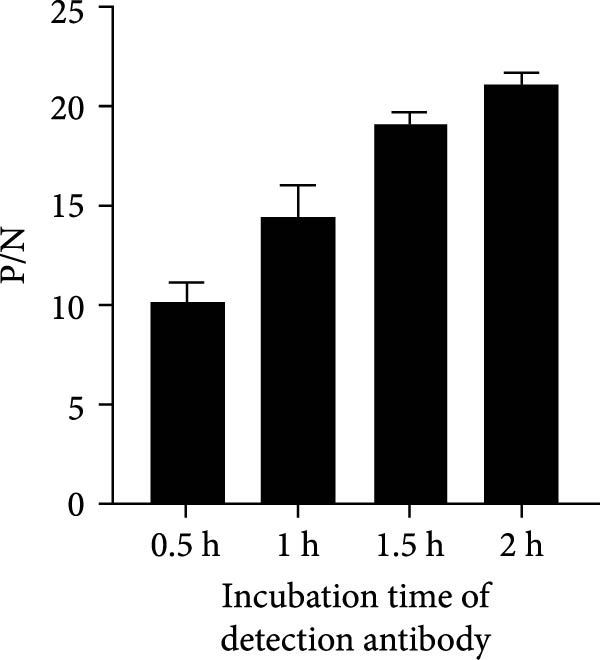
(D)

**Figure:**
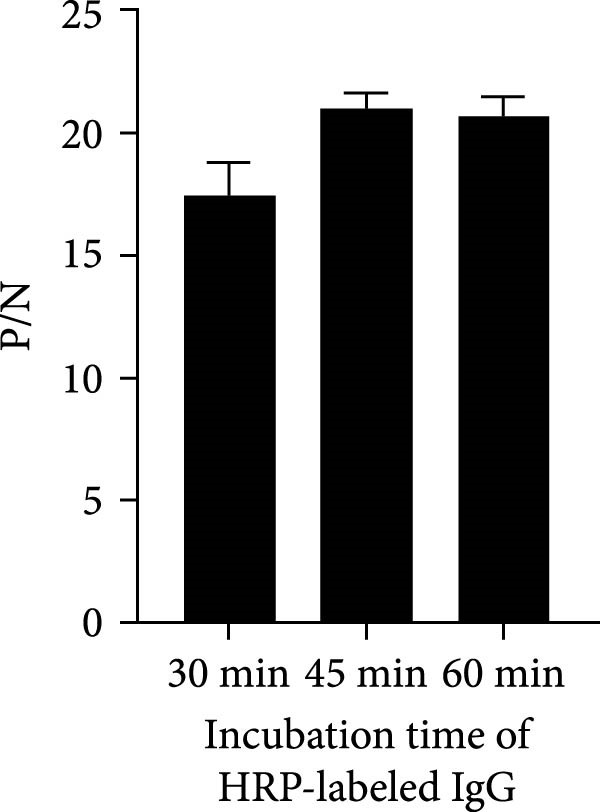
(E)

**Table 1:** A checkerboard titration was performed to determine the optimal concentration of the capture antibody 2C3 and the detection antibody pAb‐27.

pAb‐27 Dilution ratio		2C3 Concentration			
		4 μg/mL	2 μg/mL	1 μg/mL	0.5 μg/mL
1:250,000	P	1.961	1.865	**1.574**	0.197
	N	0.132	0.110	**0.078**	0.060
	P/N	14.852	16.959	**20.173**	3.311

1:500,000	P	1.2055	1.0585	0.868	0.132
	N	0.118	0.082	0.074	0.065
	P/N	10.216	12.909	11.810	2.023

1:1,000,000	P	0.7395	0.597	0.505	0.083
	N	0.133	0.082	0.066	0.045
	P/N	5.560	7.280	7.702	1.833

1:2,000,000	P	0.450	0.374	0.3065	0.065
	N	0.106	0.082	0.068	0.0515
	P/N	4.241	4.583	4.507	1.262

*Note:* Bold values represent the optimal results obtained under the corresponding experimental conditions.

### 3.5. Determination of the Cut‐Off Value

A set of 74 ENTV‐2 negative nasal swab samples and 31 ENTV‐2 positive nasal swab samples, all confirmed with WB, were tested by the p27‐acELISA. The S/P values for these samples were calculated and delineated in an interactive dot plot diagram (Figure 5A). Mean plus three standard deviations (M + 3SD) and ROC analysis are commonly used to analyze the effectiveness of a diagnostic test. Using the M + 3SD (0.0294) of the S/P values of these negative samples as the cut‐off value, the sensitivity and specificity of the p27‐acELISA were found to be 100% and 95.94%, respectively. In contrast, ROC analysis of the S/P values of all 105 nasal swab samples provided a cut‐off value of 0.1052, corresponding to a sensitivity and specificity of 100% and 100%, respectively, for the acELISA. Moreover, the AUC values showed that the acELISA is highly accurate (AUC = 1.000; 95% Cl, 1.000 – 1.000) (Figure 5B). Therefore, the cut‐off value for p27‐acELISA was determined to be 0.1052.

Figure 5Determination of the p27‐acELISA cut‐off value. (A) Interactive dot plot diagram displaying the S/P values of 74 ENTV‐2 negative nasal swab samples and 31 ENTV‐2 positive nasal swab samples. M + 3SD and ROC analysis were used to define the cut‐off value. Se, sensitivity; Sp, specificity. (B) ROC analysis of p27‐acELISA results while the AUC of the test was 1.000.

**Figure:**
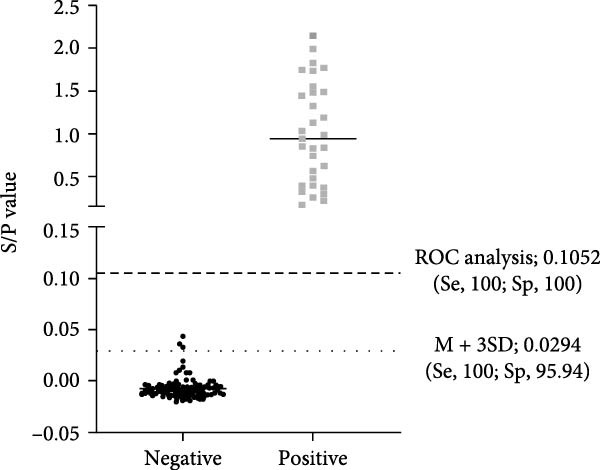
(A)

**Figure:**
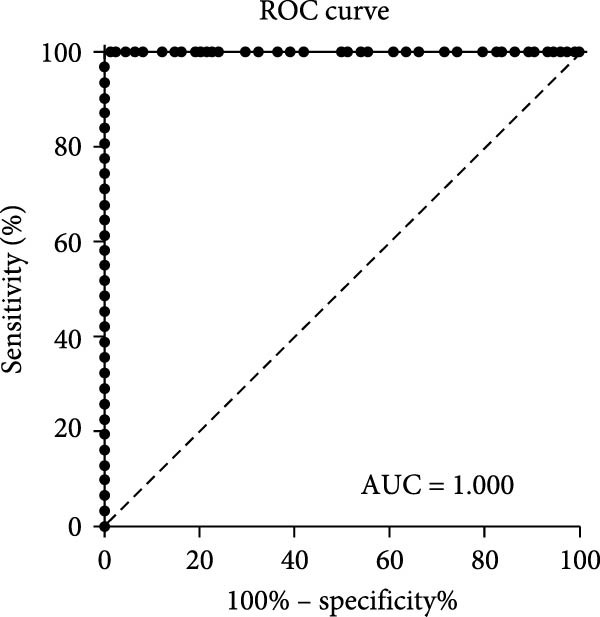
(B)

### 3.6. Specificity, Sensitivity, and Repeatability Tests

The specificity of the p27‐acELISA was evaluated using live‐attenuated or inactivated vaccines against seven common small ruminant pathogens (including FMDV, caprine *Streptococcus*, *Mycoplasma mycoides* subsp. *capri*, caprine *Escherichia coli*, ORFV, GTPV, PPRV) and the Gag proteins of ENTV‐1, JRSV, and SRLV. The Gag protein of ENTV‐2 was used as a positive control. As shown in Figure 6A, the S/P values of all eight pathogens except ENTV‐1 and JRSV were significantly lower than the cut‐off value, indicating that the proposed method did not cross‐react with these pathogens. However, the S/P values of ENTV‐1, and JSRV were higher than the cut‐off, indicating that the p27‐acELISA is capable of detecting both ENTV and JSRV.

Figure 6Analytical sensitivity and specificity of the p27‐acELISA. (A) Evaluation of the specificity of the p27‐acELISA using a panel of small ruminant pathogens as indicated. (B) Different concentrations of purified rp27 of ENTV‐1, ENTV‐2 and JSRV were used to analyze sensitivity of the p27‐acELISA. (C) Serial two‐fold dilutions of an ENTV‐2 positive nasal swab sample were used to analyze the sensitivity of the p27‐acELISA. The viral load of the initial nasal swab sample was 5.4 × 10^4^ copies/μL.

**Figure:**
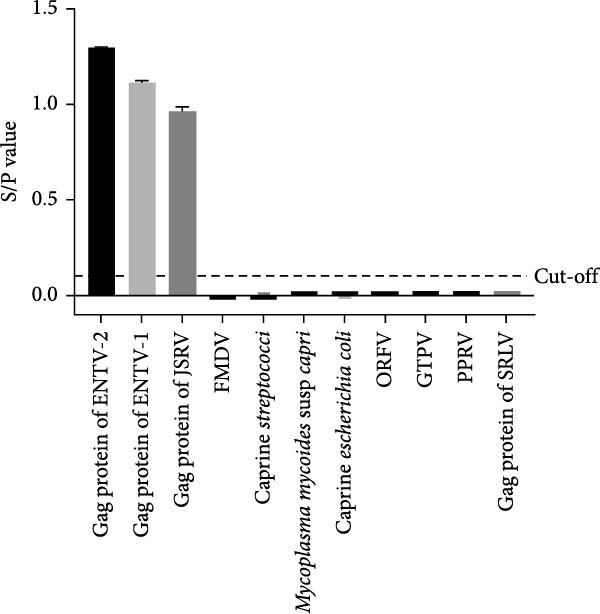
(A)

**Figure:**
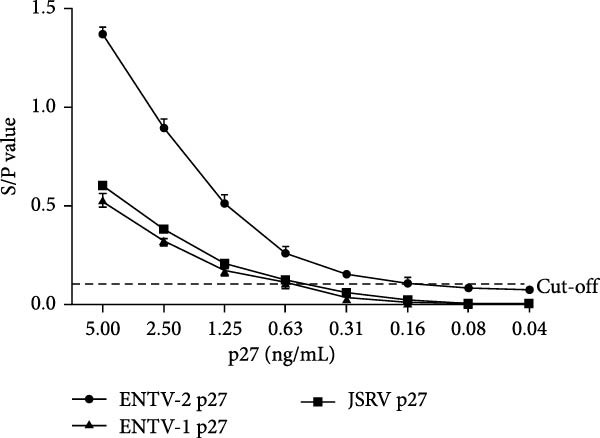
(B)

**Figure:**
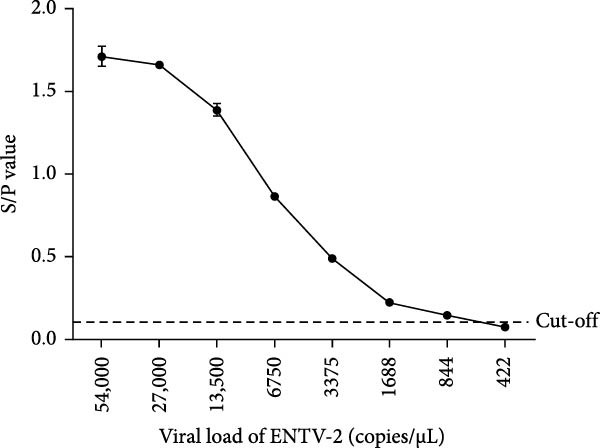
(C)

To evaluate the sensitivity of the established p27‐acELISA, the rp27 protein and one ENTV‐2 positive nasal swab sample were subjected to two‐fold serial dilution and tested using the established acELISA. The nasal swab sample was quantified using a previously reported qRT‐PCR assay [17] and had an initial viral load of 5.4 × 10^4^ copies/μL. The assay demonstrated detection limits of 0.16 ng/mL for ENTV‐2 rp27 protein and 0.63 ng/mL for ENTV‐1/JSRV rp27 protein (Figure 6B), with a sensitivity of 844 copies/μL for ENTV‐2 RNA (Figure 6C).

In the repeatability test, five different concentrations of rp27 protein and one negative control were used to evaluate the intra‐ and inter‐assay CVs of the p27‐acELISA. As detailed in Table 2, the CV for the intra‐plate repetitive and inter‐plate tests were 1.82% to 5.94% and 3.96% to 8.09%, respectively. The CV of the two repeated tests was < 10%, which confirmed that the acELISA method had good repeatability.

**Table 2:** Evaluation of p27‐acELISA reproducibility.

Concentration of rp27(ng/mL)	Intra plate			Inter plate		
	Mean	SD	CV (%)	Mean	SD	CV (%)
10	2.612	0.048	1.82	2.589	0.103	3.96
5	2.039	0.066	3.23	2.123	0.172	8.09
2.5	1.331	0.053	3.99	1.390	0.056	4.01
1.25	0.773	0.046	5.94	0.818	0.061	7.50
0.63	0.416	0.020	4.74	0.476	0.036	7.58
0	0.088	0.002	2.49	0.098	0.007	6.69

### 3.7. Agreement Between the p27‐acELISA, WB, and qRT‐PCR

A total of 191 clinical nasal swab samples were collected (105 of which were used to determine the cut‐off value in Section 3.5) and ENTV‐2 detection was performed using the p27‐acELISA, WB and qRT‐PCR (Supporting Information: Table S3). The coincidence rates of the p27‐acELISA, compared with the WB and qRT‐PCR, were 98.95% (189/191) and 96.34% (184/191), respectively (Table 3). This demonstrates the accuracy and reliability of the p27‐acELISA for clinical testing.

**Table 3:** Comparison of agreement between p27‐acELISA, WB and qRT‐PCR based on 191 field nasal swab samples.

acELISA	WB (+)	WB (−)	qRT‐PCR(+)	qRT‐PCR (−)
p27‐acELISA(+)	31	2	30	3
p27‐acELISA (−)	0	158	4	154
Agreement	98.95%		96.34%	

### 3.8. Application of the p27‐acELISA

The p27‐acELISA method developed in this study was employed to analyze 1228 nasal swab samples collected from seven provinces across China. A goat farm in Guangdong, where no goat with typical symptoms of ENT had been observed, had the highest ENTV‐2‐positive rate at 28.21% (33/117); a goat farm in Inner Mongolia, where goats with ENTV‐2 infection had previously been confirmed through pathological examination and RT‐PCR testing [15], had an ENTV‐2‐positive rate of 15.01% (59/393); a goat farm in Sichuan, where one of the goats tested (*n* = 53) had severe nasal discharge around its nose (data not shown), had an ENTV‐2‐positive rate of 15.09% (8/53). No typical ENT symptoms were observed at any of the other farms where goats or sheep were kept. As summarized in Table 4, the positivity rate for ENTV‐2 in these provinces ranged from 0.00% to 28.21%.

**Table 4:** Detection rates of ENTV‐2 in field nasal swab samples.

Area		Number of samples	Number of positive samples	Positive rate (%)	Species
Guangdong		117	33	28.21	Goat
Sichuan	Farm A	102	11	10.78	Goat
	Farm B^a^	53	8	15.09	Goat
Inner Mongolia	Farm A	79	0	0.00	Goat
	Farm B^b^	393	59	15.01	Goat
	Farm C	55	0	0.00	Goat
Heilongjiang		14	0	0.00	Goat
Anhui		38	0	0.00	Sheep
Henan		80	0	0.00	Goat
Qinghai		297	2	0.67	Goat and sheep
Total		1228	113	9.20	—

^a^One goat with severe nasal discharge was observed in this flock;

^b^Goats with nasal tumor were reported in this flock.

**Figure 7:**
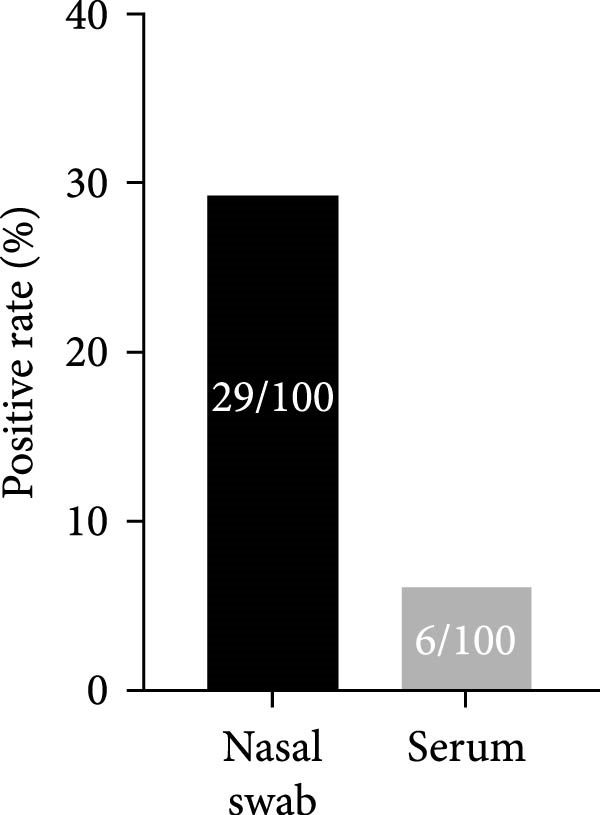
Comparison of the positive rate of ENTV‐2 in the nasal swab and serum samples from 100 goats by the acELISA.

Additionally, 100 serum samples from a goat flock in Inner Mongolia (the corresponding nasal swabs were already included in the aforementioned 1228 samples) were tested using the acELISA method. The results showed that the ENTV‐2 positive rate for serum samples was significantly lower than for nasal swabs (Figure 7 and Supporting Information: Table S4), which is consistent with previous studies [22, 23]. However, it is puzzling that six serum samples tested positive for ENTV‐2, whereas their corresponding nasal swab samples tested negative. To eliminate the possibility that the acELISA was not suitable for testing serum, we prepared a series of dilutions of an ENTV‐2‐positive nasal swab sample using negative goat serum. Subsequent acELISA testing confirmed positive results for all these ’mixed samples’, thus verifying the applicability of the assay to serum testing (Supporting Information: Table S5).

## 4. Discussion

In recent years, the continued emergence of ENTV‐2 infection in Sichuan, Fujian, Anhui, Guizhou, and Chongqing in China has caused significant economic losses to the goat breeding industry [4, 13–15, 22]. It is therefore necessary to establish a rapid, sensitive, and efficient method of detection to monitor and control the ENTV‐2 infection. There is clear evidence for the presence of ENTV‐2 in nasal exudate and tumor tissue from ENTV‐infected goats, and higher viral loads in nasal exudate have also been demonstrated [24]. In this study, we successfully developed an acELISA based on anti‐p27 antibodies for the detection of ENTV‐2 from nasal swab samples.

Retroviral CA is the most abundant viral component and is highly conserved among various retroviruses. CA is therefore a well‐established diagnostic marker for retroviruses [7–9]. In this study, we generated an mAb (2C3) and a polyclonal antibody (pAb‐p27) against ENTV‐2 CA (p27), using recombinant p27 as the immunogen. To our knowledge, 2C3 is the first reported mAb against ENTV‐2 p27. WB analysis demonstrated that both 2C3 and pAb‐p27 were able to recognize the virion‐associated p27 from nasal secretions or tumor tissue from a goat infected with ENTV‐2 [15]. Notably, the epitope sequence recognized by the 2C3 exhibits high conservation across ENTV (ENTV‐1 and ENTV‐2) and JSRV. Indeed, 2C3 and pAb‐p27 recognize eukaryoticlly expressed ENTV‐1 and JSRV Gag protein. Therefore, these two antibodies provide valuable tools for the detection of ENTV‐2, ENTV‐1, and JRSV.

Using WB analysis with these two antibodies, we observed a lack of p27 protein expression in spleen, lung, kidney, and lymphoid tissues from an ENTV‐2‐infected goat [15]. This finding is consistent with a previous study showing that tumor cells from ENTV‐2 infected goats were restricted to the nasal cavity and have not been observed in other tissues [25]. Previous immunohistochemical analysis using anti‐Env antibodies has shown that the ENTV is predominantly localized to tumor tissues, with sporadic observation in extra‐tumoral sites such as the lung and associated lymph nodes [2, 10, 11, 26–28]. In contrast, PCR‐based assays and a transcriptomic assay have occasionally detected viral DNA/RNA in a wider range of tissues other than tumor tissue [2, 13, 22, 23, 29]. Notably, endogenous *β*‐retrovirus genome sequences are widespread in the ovine and caprine genomes, with more than 85%–93% homology to the JRSV and ENTV genomes [30–32]. As a result, it remains unclear whether endogenous *β*‐retroviruses could interfere with PCR detection of ENTV. However, a previous study showed that total RNA extracted directly from blood and nasal secretion samples from healthy goats was PCR positive. Therefore, the authors concluded that it is important to remove any potential DNA contamination when performing RT‐PCR assays on ENTV [12]. Further studies are needed to determine the tissue distribution of ENTV. In this study, we confirmed via WB analysis using the 2C3 antibody that p27 is not expressed at detectable levels in the nasal mucosal cells of normal goats and sheep (Supporting Information: Figure S3). This finding illustrates the significant advantages of detecting the ENTV p27 antigen in nasal secretions for diagnosing this disease.

With the advantages of relatively low cost and high throughput, ELISA has been widely used for the detection and large‐scale monitoring of many infectious diseases. Because of the lack of circulating antibodies in animals infected with ENTV, it is not possible to diagnose ENTV infection by detecting antibodies with ELISA [33]. Therefore, the development of a double antibody sandwich ELISA for the detection of the ENTV antigen is an ideal choice, as interference by *β*‐endogenous retroviruses does not occur, as is the case with the PCR method. In this study, an acELISA was developed based on 2C3 and pAb‐27, and the reaction conditions were optimized with checkerboard titration. As there is currently no gold standard test for ENTV, a validation panel consisting of 74 ENTV‐2 negative nasal swab samples and 31 ENTV‐2 positive nasal swab samples assessed with WB was used to determine the p27‐acELISA cut‐off value, which was defined as 0.1052 of S/P based on ROC analysis. Several previously reported PCR methods for the assessment of ENTV‐2 have shown that the detection limit of conventional PCR is 300 copies/μL of RNA, and the detection limit of qRT‐PCR is between 30 and 60 copies/μL of RNA [16, 17, 22, 34]. The limit of detection of the p27‐acELISA was 0.16 ng/mL of rp27 protein and 844 copies/μL of ENTV‐2. We evaluated the performance and accuracy of the p27‐acELISA for ENTV‐2 detection by comparison with WB and qRT‐PCR. The positivity threshold of the qRT‐PCR published in the literature was set at >30 copies/μL ENTV RNA [17]. Comparative analyses showed that our p27‐acELISA had 98.95% agreement with WB and 96.34% agreement with qRT‐PCR. In addition, we also confirmed that this method did not react with eight common small ruminant pathogens: FMDV, ORFV, GTPV, PPRV, SRLV, Caprine *Streptococcus*, *Mycoplasma mycoides* subsp. *capri* and caprine *Escherichia coli*, but reacted with the Gag protein of ENTV‐1 and JSRV. These findings demonstrate that the p27‐acELISA exhibits excellent sensitivity and specificity for small ruminant (goats and sheep) *β*‐retrovirus (ENTV‐1, ENTV‐2 and JSRV). Therefore, the acELISA developed in this study can be used to diagnose ENTV‐1, ENTV‐2, and JSRV infections. While this method cannot yet distinguish between these three viruses, existing knowledge suggests that ENTV‐1 and ENTV‐2 are host‐specific, infecting sheep and goats, respectively. By contrast, JSRV primarily infects sheep, with rare cases of infection in goats reported. Coinfection by ENTV‐1 and JSRV has been reported in naturally infected sheep [35]. In addition, a potential limitation of nasal swab‐based detection is its dependence on tumor formation. Consequently, further comprehensive studies are required to enable effective monitoring during the early phases of ENTV‐2 infection.

Previously, outbreaks of ENTV‐2 infection have been reported in many areas in China, including Sichuan, Inner Mongolia, Chongqing, Shaanxi, Guangdong, Fujian, and others [3, 4, 14, 15, 17, 22, 25, 36]. However, the infection rate in these regions is not clear. In this study, 1228 clinical nasal samples collected from seven provinces in China were tested using the newly developed p27‐acELISA. We demonstrated that the infection rate of ENTV‐2 ranged from 10.78% to 28.21% in goat farms in Inner Mongolia, Sichuan, and Guangdong, suggesting that ENTV‐2 may have a wide prevalence in these areas. No ENTV‐2 positive samples were detected in goat farms in Henan and Heilongjiang. Given that these two antibodies are able to recognize the Gag proteins of ENTV‐1 and JRSV, and considering that a previous study reported that JRSV also causes nasal adenocarcinomas in sheep [37], we also tested nasal swabs collected from sheep flocks in Anhui. However, no positive samples were detected. Additionally, we tested nasal swabs from a mixed sheep and goat herd in Qinghai province and obtained two positive samples. PCR testing showed that the p27 gene sequence of these samples had the highest homology with that of ENTV‐2 (data not shown).

Currently, the diagnosis of ENT in goats and sheep primarily depends on clinical symptoms and histopathological examination. Detecting the ENTV in nasal secretions can significantly improve the efficiency of diagnosing ENT, thereby reducing the risk of the virus transmission within flocks. However, diagnosing ENT during the preclinical stage, particularly prior to tumor development, remains extremely challenging, highlighting the need for more foundational research in this area.

## 5. Conclusion

In summary, for the first time, we developed an acELISA based on two p27 antibodies for the detection of ENTV‐2. The established p27‐acELISA exhibited a high degree of specificity, sensitivity, and reproducibility and will be an important tool for large‐scale screening of ENTV‐2 infections.

## Ethics Statement

The animal experiments were approved by the Animal Care and Ethics Committees of the Harbin Veterinary Research Institute, Chinese Academy of Agricultural Sciences, under Approval Number 240703‐02‐GR and 240704‐01‐GR.

## Disclosure

All authors have reviewed the final version of the manuscript and agree to be accountable for the content and conclusions presented in this article.

## Conflicts of Interest

The authors declare no conflicts of interest.

## Author Contributions

Yang Zhao contributed to the conceptualization, designed the research, performed experiments, conducted data analysis, and wrote the original draft. Jinling Wang contributed to the conceptualization, data analysis, and constructive discussions. Xue‐Feng Wang, Jinling Wang, Qiang Liu, Jiang Wu, Qixin Huang, Bingwu Zhang, Yunze Guo, and Chang Liu collected the clinical samples. Xing Guo, Kui Guo, Weiguo Zhang, Xiaohua Ma, Xiaojun Wang, and Xue‐Feng Wang participated in the performances of the experiments. Xiaojun Wang and Xue‐Feng Wang contributed to the conceptualization, the review and editing of the writing, project administration and funding acquisition. Yang Zhao and Jinling Wang contributed equally to this article and share the first authorship. Xiaojun Wang and Xue‐Feng Wang contributed equally as corresponding author.

## Funding

This work was supported by Natural Science Foundation of Heilongjiang Province of China, TD2022C006; Xinjiang Talent Development Fund, ZZYD2023010; and Tianchi Talent Introduction Plan, IWA2023.

## Supporting Information

Additional supporting information can be found online in the Supporting Information section.

## Supporting information


**Supporting Information 1** Table S1: Information of 35 ENTV‐2 p27 amino acid sequences.

**Supporting Information 2** Table S2: List of species containing the LRPYRKKGDLSDFIRICA motif.

**Supporting Information 3** Table S3: Results of WB, qRT‐PCR and acELISA assays in 191 clinical samples.

**Supporting Information 4** Table S4: Detection of ENTV‐2 in the serum and nasal swab samples from 100 goats by the acELISA.

**Supporting Information 5** Table S5: Detection of serial dilutions of ENTV‐2 positive nasal swabs using ENTV‐2 negative goat serum by the acELISA.

**Supporting Information 6** Figure S1: Pathological diagnosis of an ENTV‐2 positive case. (A) Dissection pictures of the nasal cavity of a goat with nasal tumor. (B) Representative hamatoxylin‐eosin staining pictures of the nasal tumor. The tumor tissue is composed of closely packed, irregularly arranged glandular ducts of varying sizes. The pathological images were reproduced from reference Sheng Fu [15]: “Pathological Study and Viral Genome Sequence Analysis of Enzootic Nasal Tumor of Goat (Master’s Thesis)”, Inner Mongolia Agricultural University. Hohhot, China.

**Supporting Information 7** Figure S2: Reactivity of 2C3 or pAb‐p27 with eukaryotic ENTV‐1 or JSRV Gag protein. HEK293T cells were transfected with pcDNA‐ENTV‐1‐Gag, pcDNA‐JSRV‐Gag or empty vector (as a NC). The cells were lysed at 48 hpt for the detection of Gag using 2C3 (A) or pAb‐p27 (B) as primary antibodies.

**Supporting Information 8** Figure S3: Detection of endogenous *β*‐retrovirus in nasal mucosal cells by Western blot. (A) N1, N2, and N3 indicate nasal mucosal cell samples collected from clinically normal goats that were confirmed ENTV‐negative using the ELISA established in this study; P1, P2, and P3 indicate nasal mucosal cell samples collected from goats with nasal tumors, that were confirmed ENTV‐positive using the ELISA. (B) N4, N5, and N6 indicate nasal mucosal cell samples collected from clinically normal sheep that were confirmed ENTV‐negative using the ELISA. The recombinant protein p27 served as the positive control. *β*‐actin served as an internal control for sample loading. Nasal mucosal cells were collected from the nasal cavity via swabs. To prevent interference from exogenous retroviral components in subsequent assays, nasal swab samples were subjected to low‐speed centrifugation (400×*g*) to collect cell pellets. These pellets were then resuspended in PBS, lysed, and analyzed using Western blot with the anti‐p27 monoclonal antibody (2C3).

## Data Availability

The data that support the findings of this study are available in the Supporting Information of this article.
