# Supplementary material for: Development and Application of an Antigen Capture ELISA for the Detection of Enzootic Nasal Tumor Virus‐2
Source: Transbound Emerg Dis. 2025 Dec 19;2025:5514208. doi: 10.1155/tbed/5514208 (PMC12717442; doi:10.1155/tbed/5514208)
Supplement: Supplementary file 6 — Supporting Information 6 Figure S1: Pathological diagnosis of an ENTV‐2 positive case. (A) Dissection pictures of the nasal cavity of a goat with nasal tumor. (B) Representative hamatoxylin‐eosin staining pictures of the nasal tumor. The tumor tissue is composed of closely packed, irregularly arranged glandular ducts of varying sizes. The pathological images were reproduced from reference Sheng Fu [15]: “Pathological Study and Viral Genome Sequence Analysis of Enzootic Nasal Tumor of Goat (Master’s Thesis)”, Inner Mongolia Agricultural University. Hohhot, China. [file TBED-2025-5514208-s005.pptx]

## Slide 1
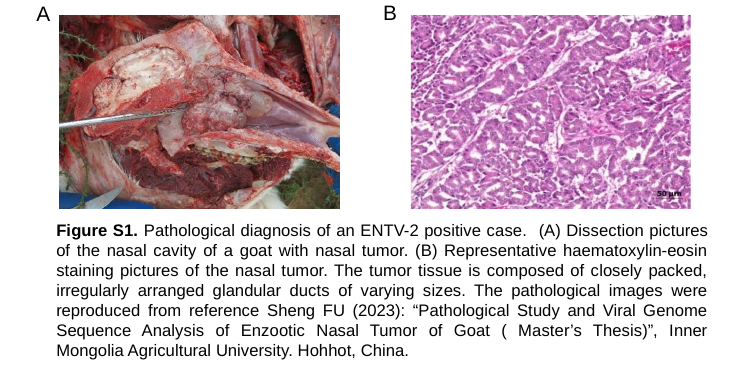

B
A
Figure S1. Pathological diagnosis of an ENTV-2 positive case. (A) Dissection pictures of the nasal cavity of a goat with nasal tumor. (B) Representative haematoxylin-eosin staining pictures of the nasal tumor. The tumor tissue is composed of closely packed, irregularly arranged glandular ducts of varying sizes. The pathological images were reproduced from reference Sheng FU (2023): “Pathological Study and Viral Genome Sequence Analysis of Enzootic Nasal Tumor of Goat ( Master’s Thesis)”, Inner Mongolia Agricultural University. Hohhot, China.
