# Supplementary material for: Development and Application of an Antigen Capture ELISA for the Detection of Enzootic Nasal Tumor Virus‐2
Source: Transbound Emerg Dis. 2025 Dec 19;2025:5514208. doi: 10.1155/tbed/5514208 (PMC12717442; doi:10.1155/tbed/5514208)
Supplement: Supplementary file 7 — Supporting Information 7 Figure S2: Reactivity of 2C3 or pAb‐p27 with eukaryotic ENTV‐1 or JSRV Gag protein. HEK293T cells were transfected with pcDNA‐ENTV‐1‐Gag, pcDNA‐JSRV‐Gag or empty vector (as a NC). The cells were lysed at 48 hpt for the detection of Gag using 2C3 (A) or pAb‐p27 (B) as primary antibodies. [file TBED-2025-5514208-s006.pptx]

## Slide 1
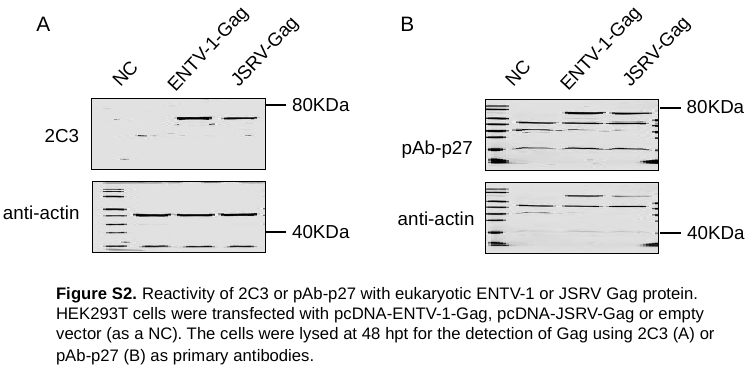

ENTV-1-Gag
JSRV-Gag
NC
80KDa
pAb-p27
anti-actin
40KDa
ENTV-1-Gag
JSRV-Gag
NC
80KDa
2C3
anti-actin
40KDa
A
B
Figure S2. Reactivity of 2C3 or pAb-p27 with eukaryotic ENTV-1 or JSRV Gag protein. HEK293T cells were transfected with pcDNA-ENTV-1-Gag, pcDNA-JSRV-Gag or empty vector (as a NC). The cells were lysed at 48 hpt for the detection of Gag using 2C3 (A) or pAb-p27 (B) as primary antibodies.
