# Supplementary material for: Development and Application of an Antigen Capture ELISA for the Detection of Enzootic Nasal Tumor Virus‐2
Source: Transbound Emerg Dis. 2025 Dec 19;2025:5514208. doi: 10.1155/tbed/5514208 (PMC12717442; doi:10.1155/tbed/5514208)
Supplement: Supplementary file 8 — Supporting Information 8 Figure S3: Detection of endogenous β‐retrovirus in nasal mucosal cells by Western blot. (A) N1, N2, and N3 indicate nasal mucosal cell samples collected from clinically normal goats that were confirmed ENTV‐negative using the ELISA established in this study; P1, P2, and P3 indicate nasal mucosal cell samples collected from goats with nasal tumors, that were confirmed ENTV‐positive using the ELISA. (B) N4, N5, and N6 indicate nasal mucosal cell samples collected from clinically normal sheep that were confirmed ENTV‐negative using the ELISA. The recombinant protein p27 served as the positive control. β‐actin served as an internal control for sample loading. Nasal mucosal cells were collected from the nasal cavity via swabs. To prevent interference from exogenous retroviral components in subsequent assays, nasal swab samples were subjected to low‐speed centrifugation (400×g) to collect cell pellets. These pellets were then resuspended in PBS, lysed, and analyzed using Western blot with the anti‐p27 monoclonal antibody (2C3). [file TBED-2025-5514208-s003.pptx]

## Slide 1
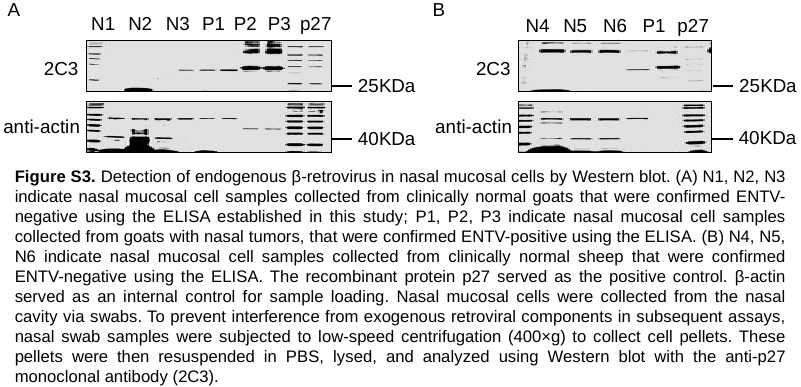

A
B
N4
N5
N6
P1
p27
2C3
25KDa
anti-actin
40KDa
p27
N1
N2
N3
P1
P2
P3
2C3
25KDa
anti-actin
40KDa
Figure S3. Detection of endogenous β-retrovirus in nasal mucosal cells by Western blot. (A) N1, N2, N3 indicate nasal mucosal cell samples collected from clinically normal goats that were confirmed ENTV-negative using the ELISA established in this study; P1, P2, P3 indicate nasal mucosal cell samples collected from goats with nasal tumors, that were confirmed ENTV-positive using the ELISA. (B) N4, N5, N6 indicate nasal mucosal cell samples collected from clinically normal sheep that were confirmed ENTV-negative using the ELISA. The recombinant protein p27 served as the positive control. β-actin served as an internal control for sample loading. Nasal mucosal cells were collected from the nasal cavity via swabs. To prevent interference from exogenous retroviral components in subsequent assays, nasal swab samples were subjected to low-speed centrifugation (400×g) to collect cell pellets. These pellets were then resuspended in PBS, lysed, and analyzed using Western blot with the anti-p27 monoclonal antibody (2C3).
